# Supplementary material for: Surface modification using heptafluorobutyric acid to produce highly stable Li metal anodes
Source: Nat Commun. 2023 May 19;14:2883. doi: 10.1038/s41467-023-38724-x (PMC10199051; doi:10.1038/s41467-023-38724-x)
Supplement: Supplementary file 1 — Supplementary Information [file 41467_2023_38724_MOESM1_ESM.pdf]

Supporting Information

Surface Modification Using Heptafluorobutyric Acid to Produce Highly Stable Li Metal Anodes

Supplementary Notes

Supplementary Note S1. The free energy for the reactions between HFA and the main components of the passivation layer (Li_2CO_3, Li_2O, LiOH). The spontaneous reaction between the main components of passivation layer (Li_2CO_3, Li_2O, LiOH) and heptafluorobutyric acid (HFA) was first investigated by theoretical calculations.

First, the free energy for the reactions between HFA and the main components of the passivation layer (Li_2CO_3, Li_2O, LiOH) were calculated by VASP5.4.1 program. VASPsol program is used to take solvation effect into consideration, and the solvent is water with a dielectric constant of 78.4.[1] KPOINTS is generated by VASPKIT program[2]. The K-Mesh is Monkhorst-Pack Scheme[3], and the resolved value is 0.025. The single molecule is in the cube cell with a side length of 25 Å. The Gibbs free energy is obtained by vibration analysis and statistical thermodynamics, and the temperature is 298.15 K. The reaction equation and calculated ΔG_r are shown in Supplementary Fig. S1. The results of the calculated reaction free energy showed that the reaction of HFA with Li_2CO_3, Li_2O, and LiOH are thermodynamically spontaneous.

Supplementary Note S2. The phase changes before and after the reaction. After the theoretical proof, experimental verification of the above reaction equation was carried out. XRD (X-ray diffraction) was employed to characterize the phase changes before and after the reaction. Li_2CO_3 (99.9%), Li_2O (99.9%) and LiOH (99.9%) were

acquired from Macklin and used as received without any further processing. According to the reaction equations, Li_2CO_3, Li_2O and LiOH were first reacted with excess HFA, and the products were dried to remove the H_2O and the residual HFA. The XRD patterns of LiOH, Li_2CO_3 and Li_2O before and after reaction with excess HFA are shown in supplementary Fig. S2. The characteristic peaks of LiOH, Li_2CO_3 and Li_2O all disappeared completely after the reaction, and the characteristic peaks of all reaction products were consistent and identical, indicating the production of HFA-Li

Supplementary Note S3. The changes in the components of the Li surface before and after HFA treatment. TOF-SIMS was used to detect changes in the components of the Li surface before and after HFA treatment. Supplementary Fig. S3a shows the components of the Li surface with sputtering time before HFA treatment. The signal of $LiO_2H_2^-$ and LiO_2H^- can be attributed to the contribution from LiOH. And the strong signals of $LiO_2H_2^-$ and LiO_2H^- suggest that the component of the passivation layer on Li surface is dominated by LiOH. An obvious signal of LiO^- can also be observed. The signal of LiO^- is from LiOH or Li_2O. And the signal of $LiCO_3^-$ usually comes from Li_2CO_3. The above results show that lithium is covered with a passivation layer composed of Li_2CO_3, LiOH, and Li_2O, and LiOH is the main component of the passivation layer. And there is also a small amount of Li_2O that is mixed in with the lithium metal. This result is consistent with the previous work. [4]

After HFA treatment, the signal coming from the component of LiOH has been significantly reduced (Supplementary Fig. S3b). And the signal of LiO^- can be attributed to the contribution of Li_2O and lithium heptafluorobutyrate. More importantly, a series of ionic fragments appear corresponding to the formation of

lithium heptafluorobutyrate. The above results directly demonstrate that HFA pre-treatment can successfully remove the surface passivation layer and construct a protective lithium fluorinatedcarboxylate interface on the Li surface. (It should be noted that TOF-SIMS can only be used to detect changes in components and cannot be used to compare the thickness of different coatings. This is because TOF-SIMS analysis has a selected sputtering property, i.e., the sputtering rate is different for different component surfaces. Typically, TOF-SIMS has a faster etch rate for organic materials than for inorganic materials.)

Supplementary Figures

$$C_3F_7COOH(aq) + LiOH(s) \rightarrow C_3F_7COOLi(aq) + H_2O(l)(1)$$

$$\Delta G_r = \mathbf{-197.068}\ \text{kJ mol}^{-1}$$

$$2C_3F_7COOH(aq) + Li_2O(s) \rightarrow 2C_3F_7COOLi(aq) + H_2O(l)(2)$$

$$\Delta G_r = \mathbf{-415.621}\ \text{kJ mol}^{-1}$$

$$2C_3F_7COOH(aq) + Li_2CO_3(s) \rightarrow 2C_3F_7COOLi(aq) + H_2O(l) + CO_2(aq)(3)$$

$$\Delta G_r = \mathbf{-278.756}\ \text{kJ mol}^{-1}$$

Supplementary Fig. S1. Chemical reaction of HFA with Li_2CO_3, Li_2O, and LiOH and the corresponding calculated reaction free energy

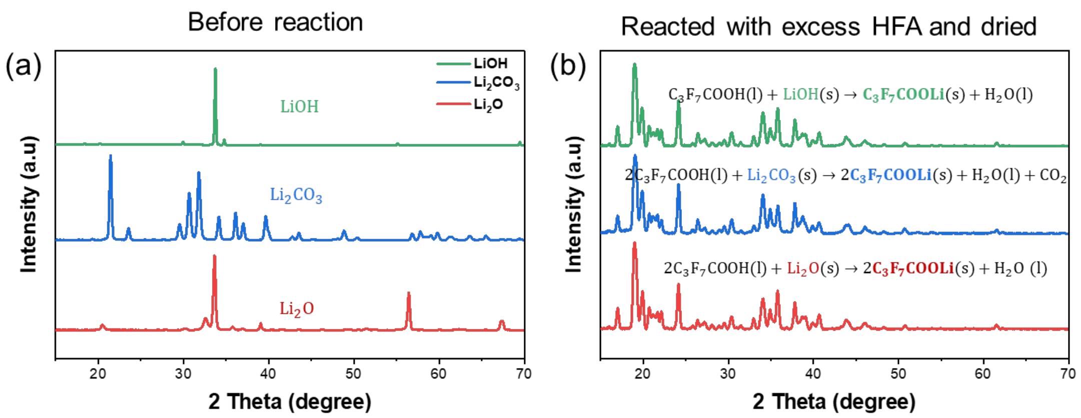

Supplementary Fig. S2. XRD patterns of LiOH, Li_2CO_3 and Li_2O before and after reaction with excess HFA.

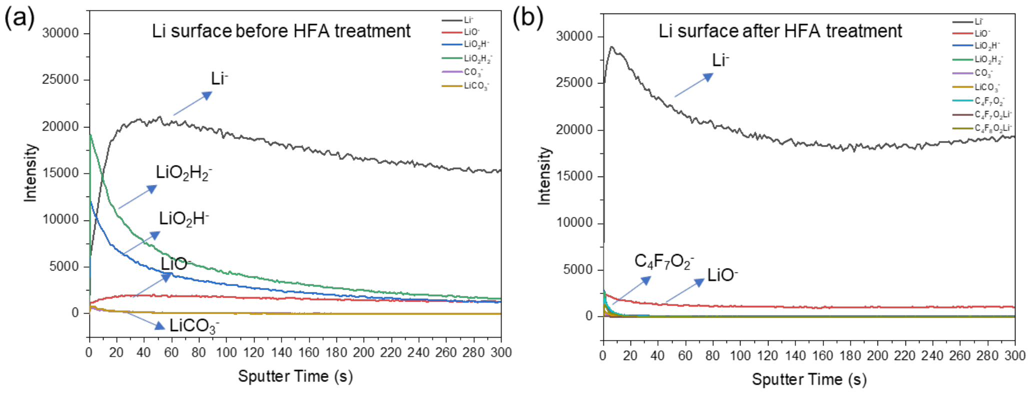

Supplementary Fig. S3. The TOF-SIMS depth sputter curves of Li surface before and after HFA treatment

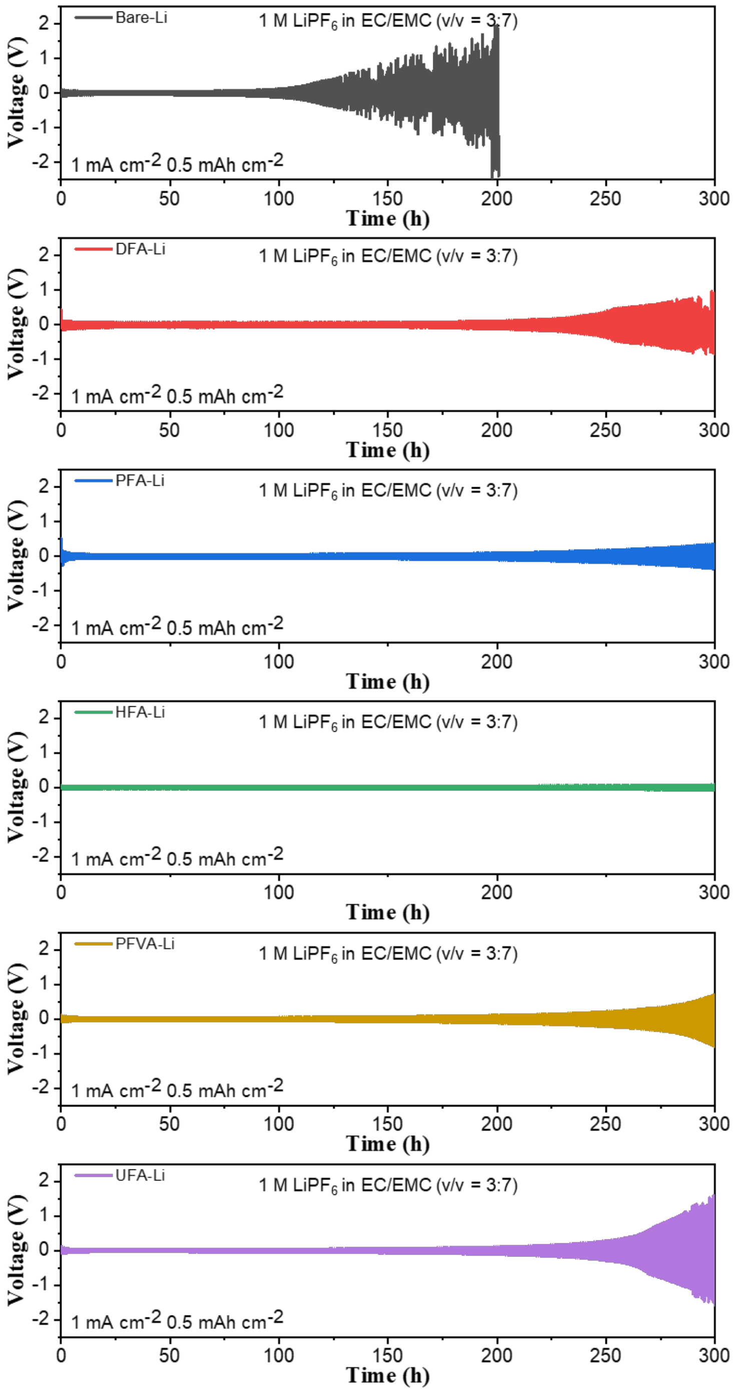

Supplementary Fig. S4. Voltage–time curves of the Li/Li symmetric cells composed of Li anodes treated with fluorinated carboxylic acids of different carbon chain lengths in a 1 M $LiPF_6$ in EC/EMC (v/v = 3:7) electrolyte at a current density of 1.0 mA cm^{-2} and a capacity of 0.5 mAh cm^{-2}. DFA-Li, PFA-Li, HFA-Li, PFVA-Li, and UFA-Li correspond to Li anodes treated with difluoroacetic acid, pentafluoropropionic acid,

heptafluorobutyric acid, perfluorovaleric acid, and undecafluorohexanoic acid, respectively.

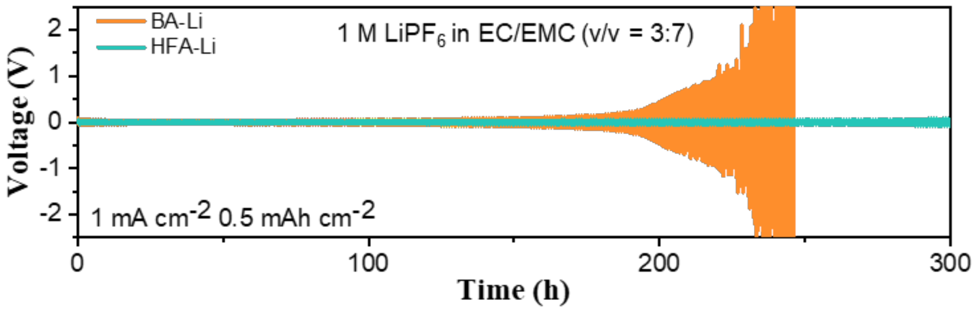

Supplementary Fig. S5. Voltage–time curves of the Li/Li symmetric composed of BA-Li and HFA-Li electrodes in a 1 M $LiPF_6$ in EC/EMC (v/v = 3:7) electrolyte at a current density of 1.0 mA cm^{-2} and a capacity of 0.5 mAh cm^{-2}.

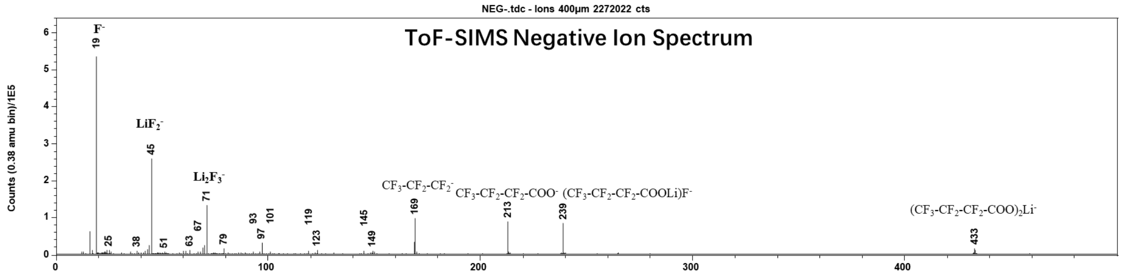

Supplementary Fig. S6. Time-of-flight secondary ion mass spectrum (TOF-SIMS) of the HFA-Li anode.

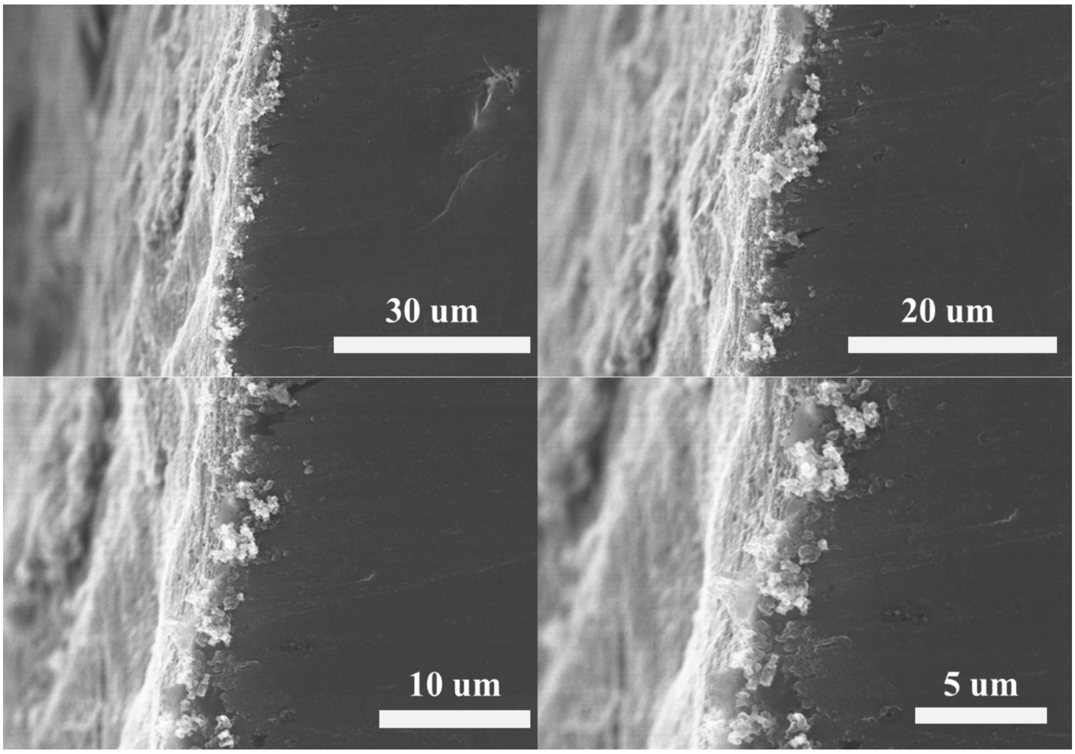

Supplementary Fig. S7. Cross-sectional SEM images of HFA-Li at different magnifications.

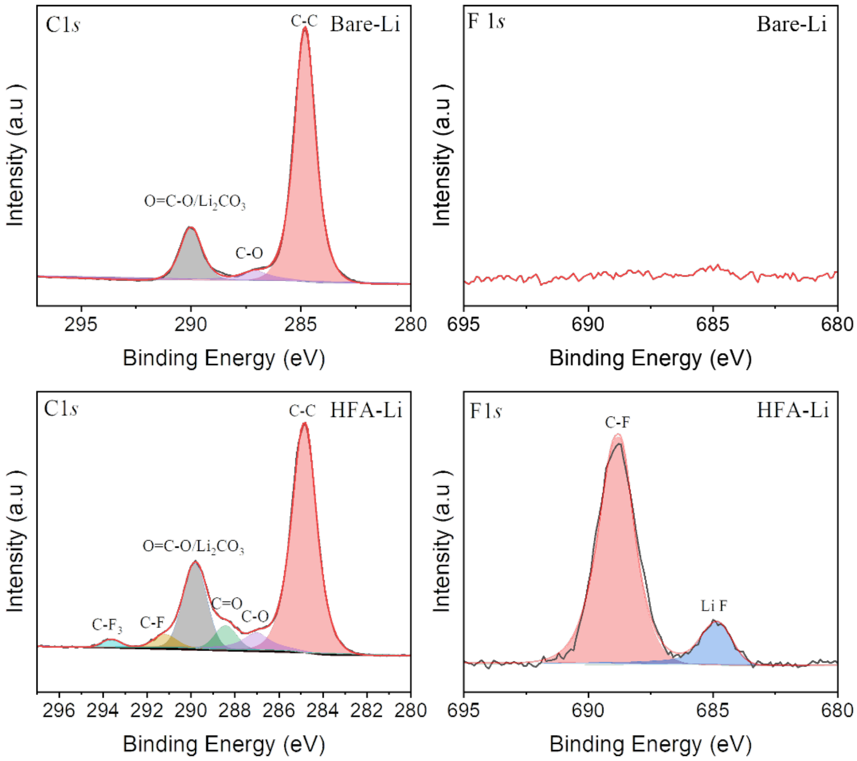

Supplementary Fig. S8. XPS spectra of C 1*s* and F 1*s* for HFA-Li and Bare Li.

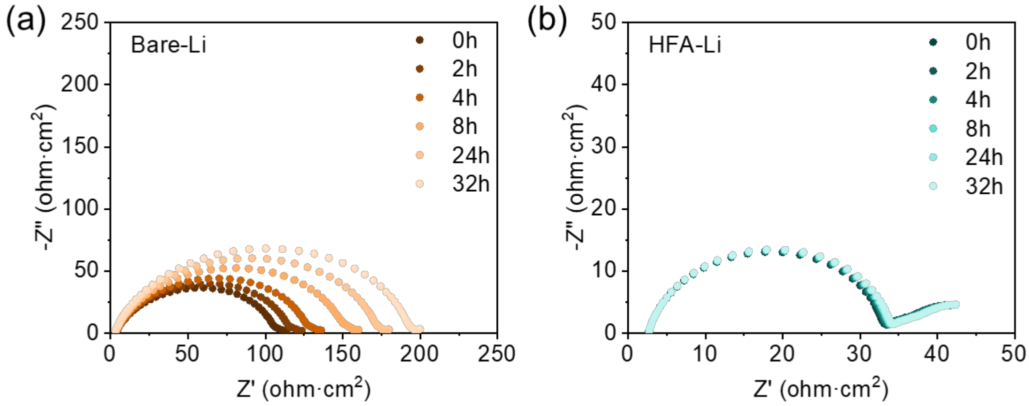

Supplementary Fig. S9. Impedance evolution of (a) Bare-Li | Bare-Li and (b) HFA-Li | HFA-Li symmetric cells over time in a 1 M $LiPF_6$ in EC/EMC (v/v = 3:7) electrolyte with 5 wt% FEC.

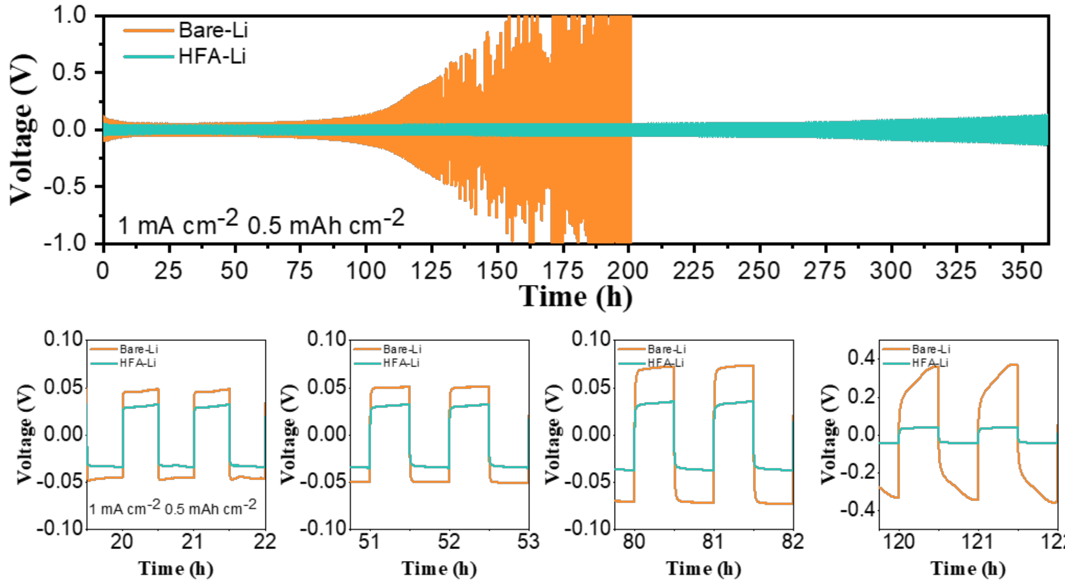

Supplementary Fig. S10. Voltage–time curves of the Li/Li symmetric cells in a 1 M $LiPF_6$ in EC/EMC (v/v = 3:7) electrolyte at a current density of 1.0 mA cm^{-2} and a capacity of 0.5 mAh cm^{-2}.

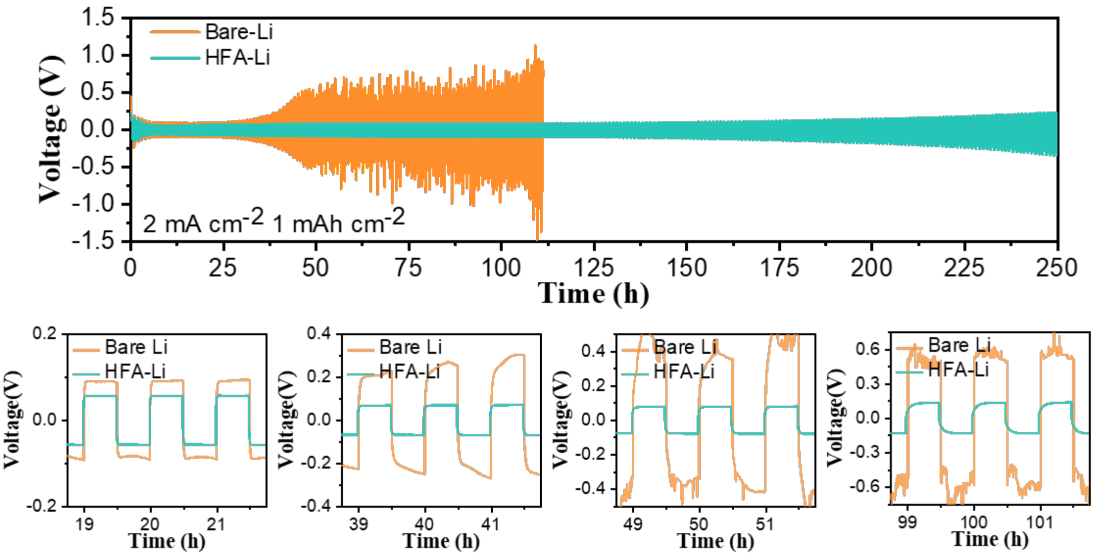

Supplementary Fig. S11. Voltage–time curves of the Li/Li symmetric cells in a 1 M $LiPF_6$ in EC/EMC (v/v = 3:7) electrolyte at a current density of 2.0 mA cm^{-2} and a capacity of 1.0 mAh cm^{-2}.

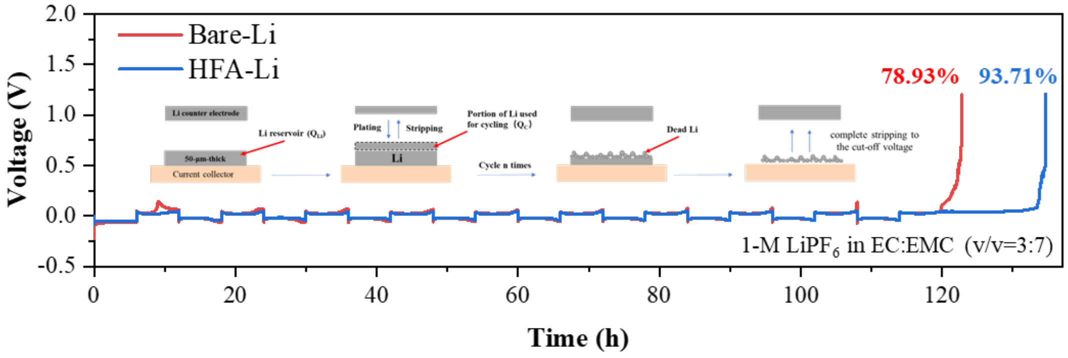

Supplementary Fig. S12. Voltage profiles of the HFA-Li and Bare-Li anodes during the Coulomb efficiency test in a 1 M $LiPF_6$ in EC/EMC (v/v = 3:7) electrolyte.

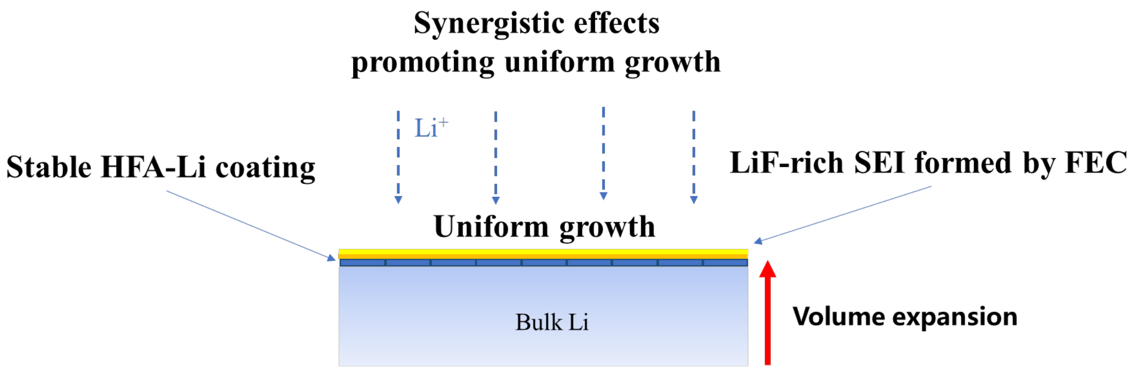

Supplementary Fig. S13. Schematic of the synergistic effect of FEC and HFA-Li on the stability of Li metal anodes.

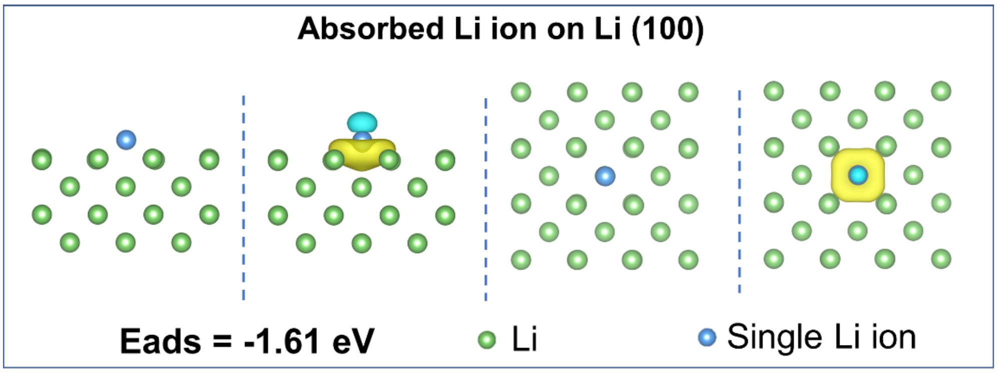

Supplementary Fig. S14. Stable configurations and corresponding charge density difference of Li ion on the surface of Li (100). The green and blue balls represent the Li, and adsorbed single Li ion, respectively. Blue and yellow regions represent charge loss and accumulation, respectively.

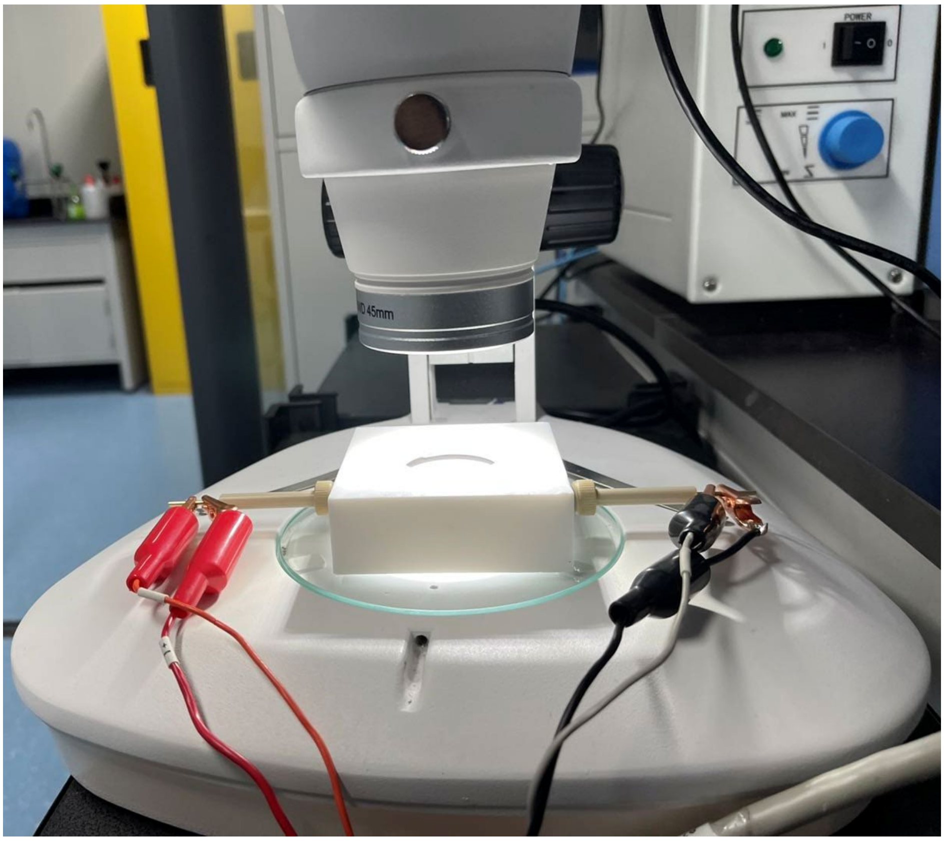

Supplementary Fig. S15. In-situ optical electrochemical cell for in-situ optical characterization.

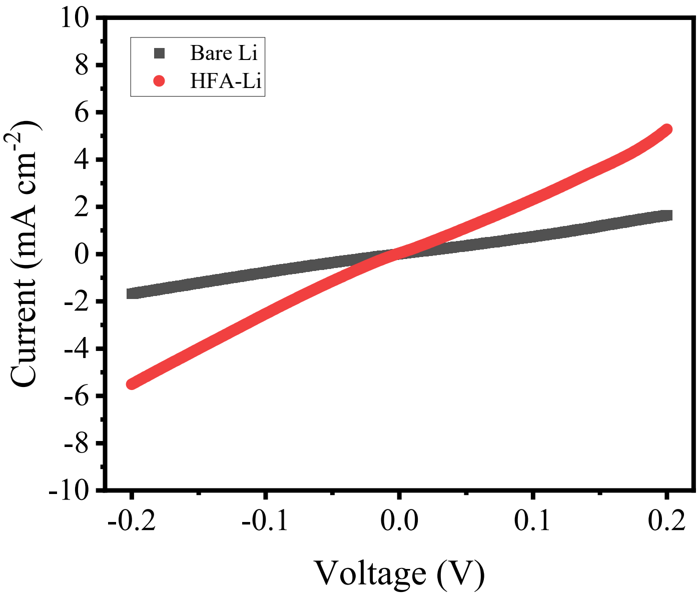

Supplementary Fig. S16. Current-voltage curves corresponding to Tafel curves for Bare-Li and HFA-Li.

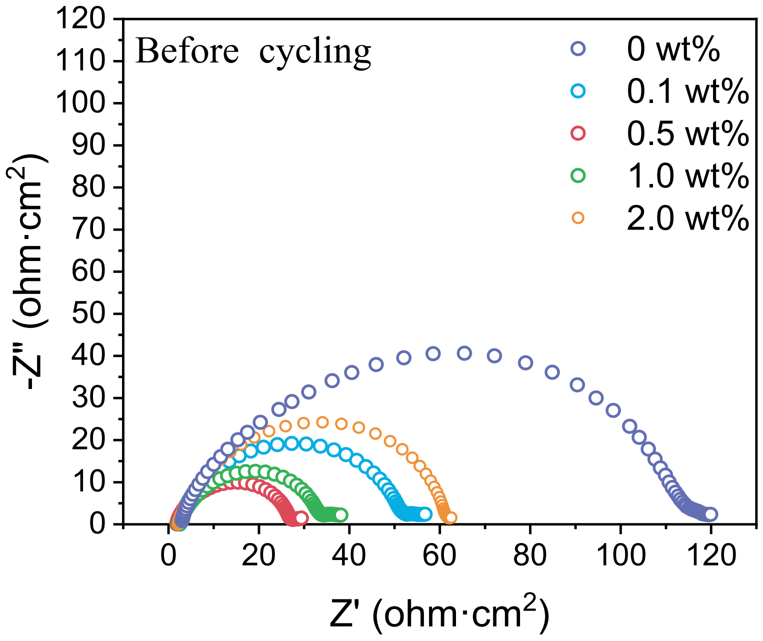

Supplementary Fig. S17. EIS plots of Li anodes after treatment with different HFA concentrations.

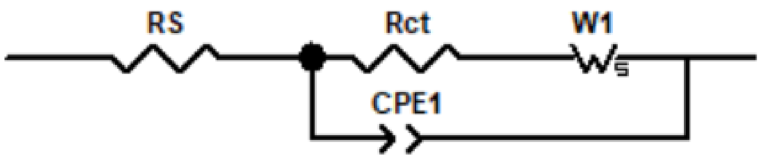

Supplementary Fig. S18. The equivalent circuit used to fit the Li/Li symmetric cells

Supplementary Tables

Supplementary Table S1. Cycling stability of HFA-Li compared with previously reported work.

Components of Artificial SEI	Electrolyte	Current density (mA cm^{-2})	Areal capacity (mAh cm^{-2})	Cycling time (h)	Number of cycles	Ref
Ag	1 M $LiPF_6$ in EC:DMC (1:2 by vol).	0.5	1.0	320	160	5
Ag	1 M $LiPF_6$ in EC:DMC (1:2 by vol).+ 10 vol% FEC	0.5	1.0	950	475	5
LiF	1.3 M $LiPF_6$ in EC:DEC (3:7 by vol).+ 5 wt% FEC	0.5	2.0	300	~38	6
LiF@Po	1.3 M $LiPF_6$ in EC:DEC (3:7 by vol).+ 5 wt% FEC	0.5	2.0	800	100	6
Li_2N	1 M $LiPF_6$ in EC/DEC (1:1)	1.0	2.0	100	25	7
Al_2O_3	1 M $LiPF_6$ in EC/DEC (1:1)	1.0	2.0	100	25	7
Li_3PO_4	1 M $LiPF_6$ in EC:DEC:DMC (1:1:1 by vol).	1.0	1.0	300	150	8
Li-PAA	1 M $LiPF_6$ in EC:DEC:DMC (1:1:1 by vol).	1.0	1.0	250	250	9
Lithium pentanoate	1 M LiTFSI in DOL/DME (1:1 by vol)	0.5	1.0	1000	250	10

PCUMA	1 m LiDFOB/EC-DMC.	0.5	1.0	1000	250	11
PVDF combined with 18-Crown-6	1 M LiTFSI in DOL:DME, (1:1 by vol) with 2 wt % $LiNO_3$).	1	1.0	140	70	12
Li alkoxides	1 M $LiPF_6$ in EC:DEC:DMC (1:1:1 by vol).	0.5	1.0	400	100	13
Li_2Se, LiCl	1 M $LiPF_6$ in EC:DEC (1:1 by vol).+ 10 vol% FEC	1.0	1.0	400	200	14
poly((N-2,2-dimethyl-1,3-dioxolane-4-methyl)-5-norbornene-exo-2,3-dicarboximide)	1 M $LiPF_6$ in EC:DMC (1:2 by vol).	0.5	1.0	300	75	15
terpolymer	1 M $LiPF_6$ in EC:EMC:DMC (1:1:1by vol).	0.5	1.0	800	200	16
PECA+$LiNO_3$	1.0 M LiPF6 in EC/DMC (1:1 by vol)	1.0	1.0	200	100	17
**HFA-Li**	**1 M $LiPF_6$ in EC:EMC (3:7 by vol)**	**1.0**	**0.5**	**350**	**350**	**This work**
**HFA-Li**	**1 M $LiPF_6$ in EC:EMC (3:7 by vol**	**2.0**	**1.0**	**200**	**200**	**This work**
**HFA-Li**	**1 M $LiPF_6$ in EC:EMC (3:7 by vol) with 5wt% FEC**	**1.0**	**0.5**	**1200**	**1200**	**This work**

Supplementary Table S2. Comparison of full cell cycling performance using different artificial SEIs.

Artificial SEI	Electrolyte	Battery Condition	Cycling Life	Ref
Adaptive "solid-liquid" interfacial protective layer (Silly Putty)	1 M LiTFSI in DOL/DME + 1wt% $LiNO_3$	5 mAh cm^{-2} deposited Li | 1.28 mAh cm^{-2} LFP	50 cycles	18
Phosphate-functionalized reduced graphene oxides	0.6 M LiTFSI, 0.4 M LiBOB, 0.4 M LiF, 0.1 M $LiNO_3$, 0.03 M $LiBF_4$, and 0.05 M $LiPF_6$ in EC/DMC + 1 wt% FEC + 2 wt% VC + 3 wt% TFEC (E-3)	50 µm Li | 4 mAh cm^{-2} NMC811	> 300 cycles	19
PDMS coating	1 M $LiPF_6$ in EC/DEC + 2 wt% VC	1 mAh cm^{-2} deposited Li | LFP	100 cycles	20
Cu_3N/SBR coating	1 M $LiPF_6$ in EC/DEC + 10 wt% FEC	10 mAh cm^{-2} deposited Li | 3 mAh cm^{-2} LTO	100 cycles	21
Li_3PO_4 SEI layer	1 M $LiPF_6$ in EC/DMC/DEC	Thick Li | 0.53 mAh cm^{-2} LFP	200 cycles	22
Li_2S coating	1 M $LiPF_6$ in EC/DEC	10 mAh cm^{-2} deposited Li | 2.5 mAh cm^{-2} LFP	150 cycles	23
Reactive polymer composite (RPC)	1 M $LiPF_6$ in EC/EMC + 2 wt% LiBOB	1.9-fold excess Li in a 3D host | 3.4 mAh cm^{-2} NMC532	200 cycles	24
Cation-Tethered Flowable Polymer	2 M LiTFSI + 2 M LiDFOB in DME	25 µm Li | 2.7 mAh cm^{-2} NMC532	70 cycles	25
LiAl-FBD coating	1 M $LiPF_6$ in EC/DEC + 10% FEC	50 µm Li | 2.6 mAh cm^{-2} NMC811	180 cycles	26
**In-situ spontaneous reaction coating HFA-Li**	**1 M $LiPF_6$ in EC/EMC+ 5 wt% FEC**	**50 µm Li | 4.0 mAh cm^{-2} NMC811**	**300 cycles**	**This work**

Supplementary Table S3. R_s and R_{ct} values of the Bare- Li anode at different temperatures

Temperature [K]	R_s [ohm·cm^2]	R_{ct} [ohm·cm^2]
303	1.719	91.1
313	1.662	52.56
323	1.802	29.25
333	1.616	12.79
343	1.528	7.5

Supplementary Table S4. R_s and R_{ct} values of the HFA-Li anode at different temperatures

Temperature [K]	R_s [ohm·cm^{-2}]	R_{ct} [ohm·cm^{-2}]
303	1.811	31.46
313	1.827	20.31
323	1.776	12.23
333	1.708	6.20
343	1.663	3.25

Supplementary References

1. Mathew K, Sundararaman R, Letchworth-Weaver K, Arias T, Hennig RG. Implicit solvation model for density-functional study of nanocrystal surfaces and reaction pathways. *The Journal of chemical physics* **140**, 084106 (2014).
2. Wang V, Xu N, Liu J-C, Tang G, Geng W-T. VASPKIT: A user-friendly interface facilitating high-throughput computing and analysis using VASP code. *Computer Physics Communications* **267**, 108033 (2021).
3. Monkhorst HJ, Pack JD. Special points for Brillouin-zone integrations. *Physical review B* **13**, 5188 (1976).
4. Otto S-K*, et al.* Storage of Lithium Metal: The Role of the Native Passivation Layer for the Anode Interface Resistance in Solid State Batteries. *ACS Applied Energy Materials* **4**, 12798-12807 (2021).
5. Peng Z*, et al.* Enhanced Stability of Li Metal Anodes by Synergetic Control of Nucleation and the Solid Electrolyte Interphase. *Advanced Energy Materials* **9**, 1901764 (2019).
6. Sun S*, et al.* Facile ex situ formation of a LiF–polymer composite layer as an artificial SEI layer on Li metal by simple roll-press processing for carbonate electrolyte-based Li metal batteries. *Journal of Materials Chemistry A* **8**, 17229-17237 (2020).
7. Park K, Goodenough JB. Dendrite-Suppressed Lithium Plating from a Liquid Electrolyte via Wetting of Li_3N. *Advanced Energy Materials* **7**, 1700732 (2017).
8. Wang L, Wang Q, Jia W, Chen S, Gao P, Li J. Li metal coated with amorphous Li_3PO_4 via magnetron sputtering for stable and long-cycle life lithium metal batteries. *Journal of Power Sources* **342**, 175-182 (2017).
9. Li N-W*, et al.* A Flexible Solid Electrolyte Interphase Layer for Long-Life Lithium Metal Anodes. *Angewandte Chemie International Edition* **57**, 1505-1509 (2018).
10. Kang D*, et al.* In-situ organic SEI layer for dendrite-free lithium metal anode. *Energy Storage Materials* **27**, 69-77 (2020).
11. Hu R*, et al.* A Polymer-Reinforced SEI Layer Induced by a Cyclic Carbonate-Based Polymer Electrolyte Boosting 4.45 V $LiCoO_2$ /Li Metal Batteries. *Small* **16**, e1907163 (2020).
12. Dong H*, et al.* High lithium-ion conductivity polymer film to suppress dendrites in Li metal batteries. *Journal of Power Sources* **423**, 72-79 (2019).
13. Kang D*, et al.* Rearrange SEI with artificial organic layer for stable lithium metal anode. *Energy Storage Materials* **24**, 618-625 (2020).
14. Lee D*, et al.* Stable artificial solid electrolyte interphase with lithium selenide and lithium chloride for dendrite-free lithium metal anodes. *Journal of Power Sources* **506**, 230158 (2021).
15. Gao Y, Zhao Y, Li YC, Huang Q, Mallouk TE, Wang D. Interfacial Chemistry Regulation via a Skin-Grafting Strategy Enables High-Performance Lithium-Metal Batteries. *J Am Chem Soc* **139**, 15288-15291 (2017).

16. Wang X*, et al.* Simultaneously Regulating Lithium Ion Flux and Surface Activity for Dendrite-Free Lithium Metal Anodes. *ACS Appl Mater Interfaces* **11**, 5159-5167 (2019).
17. Hu Z*, et al.* Poly(ethyl α-cyanoacrylate)-Based Artificial Solid Electrolyte Interphase Layer for Enhanced Interface Stability of Li Metal Anodes. *Chemistry of Materials* **29**, 4682-4689 (2017).
18. Liu K*, et al.* Lithium Metal Anodes with an Adaptive "Solid-Liquid" Interfacial Protective Layer. *J Am Chem Soc* **139**, 4815-4820 (2017).
19. Kim MS*, et al.* Langmuir–Blodgett artificial solid-electrolyte interphases for practical lithium metal batteries. *Nature Energy* **3**, 889-898 (2018).
20. Zhu B*, et al.* Poly(dimethylsiloxane) Thin Film as a Stable Interfacial Layer for High-Performance Lithium-Metal Battery Anodes. *Adv Mater* **29**, (2017).
21. Liu Y*, et al.* An Artificial Solid Electrolyte Interphase with High Li-Ion Conductivity, Mechanical Strength, and Flexibility for Stable Lithium Metal Anodes. *Adv Mater* **29**, 1605531 (2017).
22. Li NW, Yin YX, Yang CP, Guo YG. An Artificial Solid Electrolyte Interphase Layer for Stable Lithium Metal Anodes. *Adv Mater* **28**, 1853-1858 (2016).
23. Chen H*, et al.* Uniform High Ionic Conducting Lithium Sulfide Protection Layer for Stable Lithium Metal Anode. *Advanced Energy Materials* **9**, 1900858 (2019).
24. Gao Y*, et al.* Polymer-inorganic solid-electrolyte interphase for stable lithium metal batteries under lean electrolyte conditions. *Nat Mater* **18**, 384-389 (2019).
25. Huang Z, Choudhury S, Gong H, Cui Y, Bao Z. A Cation-Tethered Flowable Polymeric Interface for Enabling Stable Deposition of Metallic Lithium. *J Am Chem Soc* **142**, 21393-21403 (2020).
26. Yu Z*, et al.* A Solution-Processable High-Modulus Crystalline Artificial Solid Electrolyte Interphase for Practical Lithium Metal Batteries. *Advanced Energy Materials* **12**, 2201025 (2022).
