# Supplementary material for: Surface modification using heptafluorobutyric acid to produce highly stable Li metal anodes
Source: Nat Commun. 2023 May 19;14:2883. doi: 10.1038/s41467-023-38724-x (PMC10199051; doi:10.1038/s41467-023-38724-x)
Supplement: Supplementary file 3 — Description of Additional Supplementary Files [file 41467_2023_38724_MOESM3_ESM.pdf]

Description of Additional Supplementary Files

File Name: Supplementary Movie 1
Description: In-situ optical Li deposition Movie of Bare-Li.

File Name: Supplementary Movie 2
Description: In-situ optical Li deposition Movie of HFA-Li.
